# Nanovaccines empowering CD8^+^ T cells: a precision strategy to enhance cancer immunotherapy

**DOI:** 10.7150/thno.107856

**Published:** 2025-02-10

**Authors:** Yunfan Lin, Pei Lin, Rongwei Xu, Xu Chen, Ye Lu, Jiarong Zheng, Yucheng Zheng, Zihao Zhou, Zizhao Mai, Xinyuan Zhao, Li Cui

**Affiliations:** 1 Stomatological Hospital, School of Stomatology, Southern Medical University, Guangzhou, 510280, Guangdong, China.; 2 Department of Dentistry, The First Affiliated Hospital, Sun Yat-sen University, Guangzhou 510080, China.; 3 School of Dentistry, University of California, Los Angeles, Los Angeles, 90095, CA, USA.

**Keywords:** cancer immunotherapy, nanovaccine, CD8^+^ T cell, tumor microenvironment

## Abstract

Cancer immunotherapy leveraging nanovaccines represents a cutting-edge frontier in precision medicine, specifically designed to potentiate CD8^+^ T cell-based immunotherapy. This review thoroughly delineates the evolving landscape of cancer nanovaccine development, emphasizing their advantageous role in modulating the immunosuppressive tumor microenvironment (TME) to enhance CD8^+^ T cell efficacy. We critically analyze current innovations in nanovaccine design, focusing on their capacity to deliver tumor antigens and immunostimulatory adjuvants effectively. These nanovaccines are engineered to overcome the physical and immunological barriers of the TME, facilitating the robust activation and proliferation of CD8^+^ T cells. Challenges such as delivery efficacy, safety, and scalable manufacturing are discussed, alongside future prospects which include the potential of developing specific biomaterial approaches to provide durable antitumor immunity. This comprehensive analysis not only underscores the transformative potential of cancer nanovaccines in enhancing CD8^+^ T cell responses but also highlights the critical need for advanced solutions to overcome the complex interplay of factors that limit the efficacy of current immunotherapies.

## Introduction

Cancer immunotherapy has emerged as a transformative approach in oncology, aiming to harness and enhance the body's immune system to combat malignant cells. Among the various immunotherapeutic strategies, activating or promoting CD8^+^ T cell responses has shown remarkable efficacy in targeting and eliminating tumor cells. CD8^+^ T cells, also known as cytotoxic T lymphocytes (CTLs), are critical for recognizing and destroying cancer cells. However, the effectiveness of this approach is often limited by the tumor microenvironment (TME), which can suppress immune activity and hinder CD8^+^ T cell infiltration and function 1. The TME is a complex and dynamic ecosystem composed of various cell types, including cancer cells, immune cells, fibroblasts, and endothelial cells, as well as extracellular matrix components and soluble factors. This environment can be highly immunosuppressive that inhibit the activation and function of effector T cells. Additionally, the TME often exhibits physical barriers such as dense stromal networks and abnormal vasculature that impede the efficient trafficking and infiltration of CD8^+^ T cells into the tumor core 2.

Addressing these challenges is critical for enhancing the efficacy of CD8^+^ T cell-based immunotherapies. Distinct strategies, such as nanovaccines, are being developed to overcome the immunosuppressive barriers of the TME and enhance the activation, infiltration, and function of CD8^+^ T cells. Nanovaccines utilize the unique capabilities of nanotechnology to enhance the delivery of tumor antigens and adjuvants, ensuring efficient uptake by antigen-presenting cells (APCs) and robust activation of CD8^+^ T cells. They can modulate the TME by incorporating immune-modulating agents, counteracting immunosuppressive conditions, and creating a more favorable environment for T cell activity. Additionally, nanovaccines provide sustained stimulation of the immune system through controlled release mechanisms, ultimately promoting a more robust and durable antitumor response 3, 4.

In this review, the following key aspects regarding the utilization of cancer nanovaccines for enhancing CD8^+^ T cell-based immunotherapy are highlighted. Firstly, the current landscape of cancer vaccine development is analyzed, emphasizing the advantages in eliciting immune responses and addressing the limitations in practical applications. Importantly, an in-depth and critical review of cancer nanovaccines is provided, detailing their fundamental components and advantages, and categorizing them based on elements crucial to the action on CD8^+^ T cells. Additionally, the potential of cancer nanovaccines to modify the immunosuppressive conditions within the TME, thereby favoring the activation and proliferation of CD8^+^ T cells, is underscored. Finally, the current challenges and future prospects for using cancer nanovaccines to enhance CD8^+^ T cell-based immunotherapy are discussed.

## Key factors influencing CD8⁺ T cell efficacy within the TME

CD8^+^ T cells, central to the adaptive immune response, originate from hematopoietic stem cells in the bone marrow and undergo maturation in the thymus where they differentiate into functionally distinct subsets based on their T cell receptor specificity and the expression of surface markers 5. Their initial activation occurs upon recognition of peptide antigens presented by MHC class I molecules on the surface of APCs. Once activated, CD8^+^ T cells proliferate and differentiate into effector cells that can recognize and kill. Additionally, they can trigger apoptosis in cancer cells through interactions between Fas and Fas ligand 6. Notably, the effectiveness of CD8^+^ T cells in cancer is markedly determined by their ability to persist as memory cells after the initial immune response, providing long-term surveillance and protection against tumor recurrence. For instance, CD8^+^ T cells with a tissue-resident memory phenotype within the tumor play a crucial role in local immunity and responses to immune checkpoint inhibitors 7.

**Figure 1:**
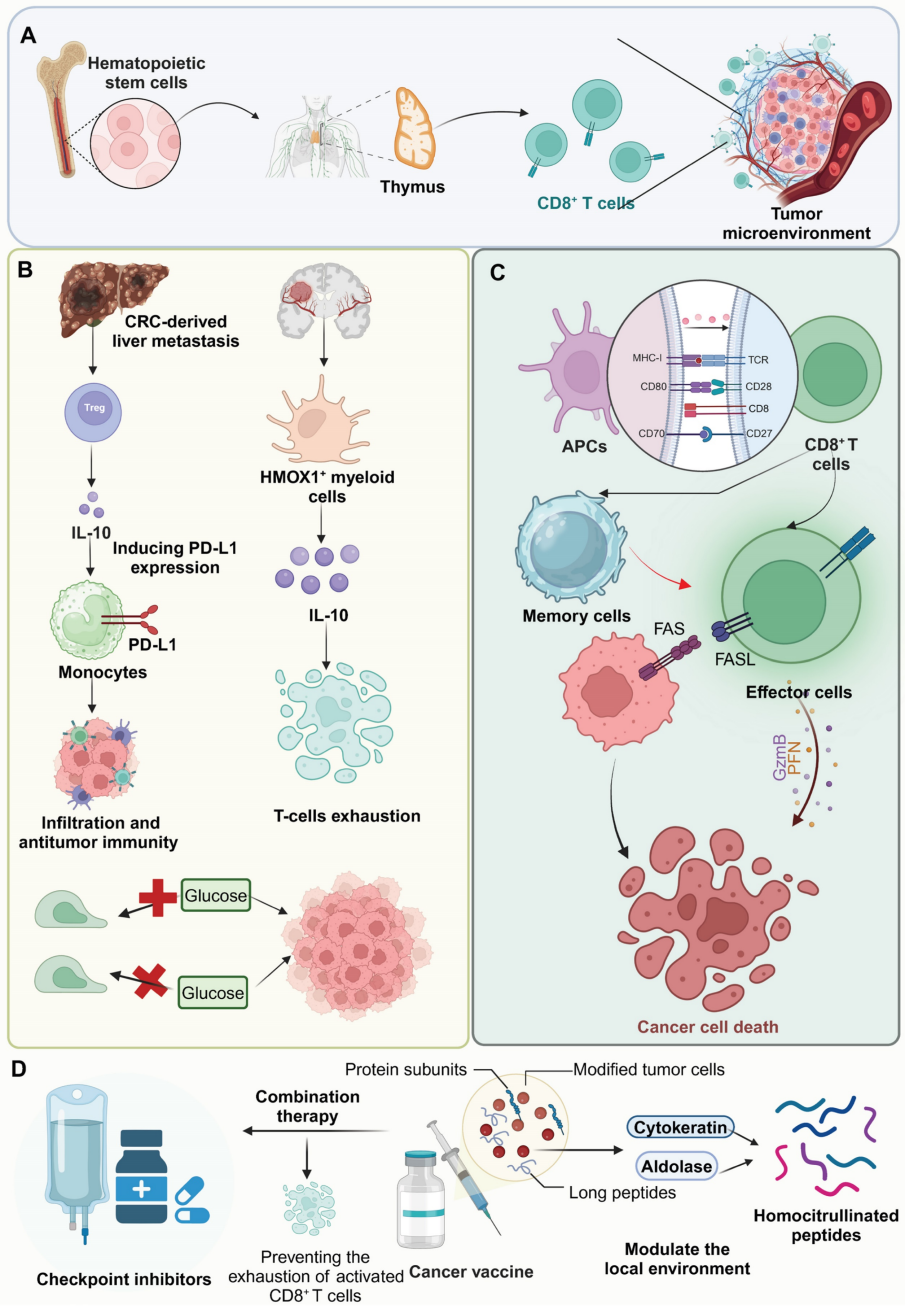
** Mechanisms of T-cell activation, immune suppression, and therapeutic strategies in the TME.** (A) Hematopoietic stem cells from bone marrow differentiate into T cells in the thymus, with mature CD8^+^ T cells migrating to the immunosuppressive TME. (B) In colorectal cancer liver metastases, Tregs and HMOX1^+^ myeloid cells secrete IL-10, promoting PD-L1 expression on monocytes and T-cell exhaustion, while glucose availability affects tumor growth. (C) T-cell activation occurs when antigen-presenting cells present tumor-associated antigens via MHC-I with co-stimulatory signals, leading to effector T-cell differentiation and cancer cell death through perforin/granzyme B release or Fas/FasL pathways. (D) Therapeutic strategies include checkpoint inhibitors to enhance CD8^+^ T-cell activation and prevent exhaustion, and cancer vaccines with protein subunits and modified tumor cells to modulate the TME and improve recognition of homocitrullinated peptides by effector T cells, collectively enhancing anti-tumor immune responses.

The TME is a complex network that critically impedes CD8^+^ T cell function and efficacy through a series of dynamic and interrelated mechanisms. Foremost among these are the activities of various immunosuppressive cells such as regulatory T cells (Tregs), myeloid-derived suppressor cells (MDSCs), and tumor-associated macrophages (TAMs). These cells exert profound inhibitory effects on CD8^+^ T cells by secreting cytokines, which not only suppress effector functions but also upregulate inhibitory checkpoint molecules such as PD-1 on T cells and PD-L1 on APCs. For instance, IL-10-releasing HMOX1^+^ myeloid cells, localized to mesenchymal-like regions in glioblastomas, drive T-cell exhaustion and contribute to the immunosuppressive TME 8. Similarly, in colorectal cancer-derived liver metastases, Treg-derived IL-10 induces PD-L1 expression in monocytes, reducing CD8^+^ T-cell infiltration and antitumor immunity 9. Concurrently, these immunosuppressive cells deplete critical nutrients and produce reactive molecules, leading to a hostile metabolic environment. This nutritional competition is exacerbated by the rapid consumption of glucose and amino acids by tumor cells, leaving CD8^+^ T cells starved and metabolically exhausted. Tumor cells outcompete CD8^+^ T cells for methionine by expressing high levels of the transporter SLC43A2, disrupting T cell methionine metabolism. This reduces intracellular methionine and S-adenosylmethionine leading to loss of H3K79me2, decreased STAT5 expression, and impaired T cell immunity 10. Moreover, the hypoxic conditions prevalent within the TME further contribute to T cell dysfunction by stabilizing hypoxia-inducible factors, which shift T cell metabolism towards less efficient pathways, thus promoting an exhausted phenotype. Hypoxia modulates CD8^+^ T cell exhaustion by driving the differentiation of PD-1^+^ TIM-3^+^ CXCR5^+^ terminally exhausted-like subsets while reducing progenitor-like populations, which is mediated by hypoxia-induced VEGF-A 11. Additionally, the dense ECM and high interstitial pressure physically restrict T cell infiltration and impede their mobility and access to tumor cells. In undifferentiated pleomorphic sarcoma, dense ECM deposition, driven by YAP1-mediated collagen VI accumulation, impedes CD8^+^ T cell infiltration and function 12. Notably, the altered chemokine and cytokine milieu within the TME also misguides T cells away from the tumor core, diminishing effective immune surveillance and cytotoxic activity. High tumor cell-intrinsic EZH2 expression in esophageal squamous cell carcinoma drives an immune-desert TME by disrupting CXCL9-mediated CD8^+^ T cell recruitment via NF-κB dysregulation. This, along with impaired dendritic cell (DC) maturation due to reduced VEGFC secretion, limits CD8^+^ T cell infiltration and promotes immune evasion 13** (Figure 1)**.

## Overview of cancer nanovaccines

**Figure 2:**
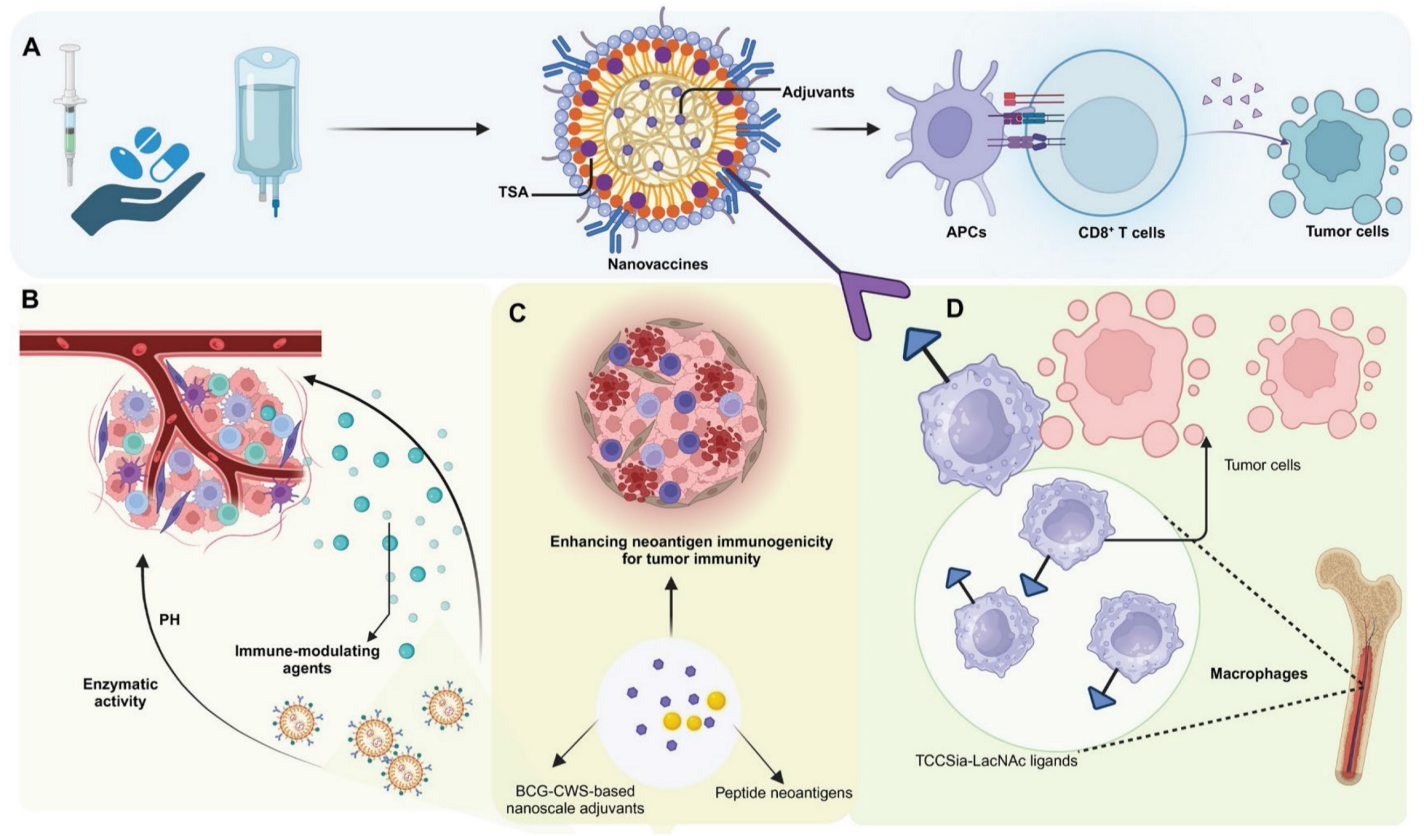
** Nanovaccine strategies and immune modulation for enhancing anti-tumor responses in the TME.** (A) Nanovaccines incorporating TSA and adjuvants are administered, leading to enhanced antigen presentation by APCs. This results in the activation of CD8^+^ T cells, which subsequently target and destroy tumor cells. (B) pH-sensitive and enzymatically-responsive nanocarriers modulate the TME by releasing immune-modulating agents in response to specific tumor conditions, facilitating immune cell infiltration into the tumor site. (C) BCG-CWS-based nanoscale adjuvants and peptide neoantigens are employed to enhance neoantigen immunogenicity and tumor-specific immune responses, improving the recognition of cancer cells by the immune system. (D) TCCSia-LacNAc ligands are utilized to reprogram macrophages within the TME, promoting their anti-tumor activity and ability to phagocytose tumor cells. These combined strategies aim to overcome immunosuppression in the TME and potentiate anti-tumor immunity through multiple mechanisms.

An ideal cancer vaccine is engineered to provoke a potent, specific, and lasting immune response, targeting the complexities and inherent heterogeneities of cancer with precision. Crucially, it incorporates tumor-specific antigens or neoantigens, which are unique to cancer cells and absent in normal tissues, thereby minimizing the risk of autoimmune reactions and focusing the immune attack on malignant cells 14, 15. To enhance this response, the vaccine also includes effective adjuvants that stimulate the innate immune system, facilitating the robust presentation of antigens and the activation of T cells, essential for initiating a strong and specific immune response 16. Additionally, the formulations of these vaccines are precisely designed to stabilize the active ingredients, enhance their delivery, and maintain the bioactivity of the antigens and adjuvants until they reach their target. Equally critical is the selection of an effective delivery system—such as viral vectors, liposomes, or nanosystems—capable of efficiently delivering antigens and adjuvants into host cells for effective uptake and processing 17-19. Together, these elements enable precise tumor targeting while fostering durable immunological memory, offering sustained protection against tumor recurrence and progression 20 **(Figure 2)**.

The design of nanoparticle vaccines requires precise engineering of their physicochemical properties to effectively address the complex challenges presented by the TME. Particle size is crucial, as nanoparticles smaller than 100 nm are particularly effective for tumor targeting. These smaller particles are able to penetrate the tumor's leaky vasculature more efficiently and avoid uptake by the normal tissue endothelium. This optimal sizing enhances their residence time in the bloodstream, thereby maximizing their accumulation at the tumor site 21. In addition, surface charge plays a significant role in how nanoparticle vaccines interact with cellular and extracellular components within the TME. Positively charged nanoparticles facilitate enhanced cellular uptake due to their strong electrostatic interactions with the negatively charged components of cell membranes. This promotes the efficient internalization and delivery of therapeutic agents into tumor cells. However, this positive charge also poses a risk of rapid clearance from the bloodstream through opsonization. Conversely, nanoparticles with neutral or slightly negative charges tend to have longer circulation times due to their reduced interactions with blood proteins, which improves their passive targeting capabilities 22. Furthermore, the inclusion of targeting ligands in nanoparticle vaccines significantly improves their specificity and delivery efficacy. These ligands are specifically designed to bind to receptors or other molecules that are overexpressed on tumor cells or immune-modulatory cells within the TME. This targeted delivery approach ensures the direct administration of active compounds to these cells, maximizing therapeutic efficacy while minimizing potential side effects 23.

Building on the physicochemical advancements discussed above, the strategic design of nanovaccines focuses on overcoming immunosuppressive pathways within the TME to enhance CD8^+^ T cell responses. Nanoparticles can be engineered to deliver specific molecular inhibitors that disrupt the functions of immunosuppressive cells. For instance, encapsulating CSF1-R and IDO inhibitors, the tumor acidity-responsive nanovaccine sheds its PEG shell under acidic conditions, reducing size and increasing positive charge to enhance tumor penetration. It reprograms TAMs and remodels the TME into a T cell-favorable environment, effectively inhibiting tumor growth in E.G7-OVA models 24. Additionally, incorporating immune-modulating adjuvants that transform the TME from an immunosuppressive to an immunostimulatory state is crucial. Lipid-based immunostimulatory nanoparticles, encapsulating TLR4 and STING agonists, synergistically enhance the efficacy of anti-PD1 therapy in aggressive murine tumor models. These nanoparticles significantly activate CD8^+^ T cells, leading to substantial tumor clearance and the development of protective immunological memory. Such inspiring designs not only counteract the local suppressive effects but also promote a more robust infiltration and persistence of activated CD8^+^ T cells, thereby significantly enhancing the efficacy of the immunotherapy 25.

## Mechanisms and types of nanovaccine adjuvants

### Mechanism of adjuvants

Adjuvants are crucial components of nanovaccines as they activate the innate immune system, thereby enhancing antigen presentation and amplifying the overall immune response 26, 27. Mechanistically, vaccine adjuvants enhance immune responses by targeting pattern recognition receptors (PRRs) such as toll-like receptors (TLRs), NOD-like receptors (NLRs), RIG-I-like receptors (RLRs), and C-type lectin receptors (CLRs) on APCs. Activation of these receptors initiates intracellular signaling cascades that lead to the upregulation of pro-inflammatory cytokines, co-stimulatory molecules, and antigen presentation pathways 28, 29. Surface TLRs, such as TLR2 and TLR4, activate MyD88-dependent pathways, stimulating NF-κB and promoting the production of cytokines like IL-6, TNF-α, and IL-12, which support Th1/Th2 polarization and APC maturation 30. Endosomal TLRs, including TLR3 and TLR9, trigger TRIF-dependent pathways, leading to IRF3 and IRF7 activation and type I IFN production, critical for CD8^+^ T cell responses 31. Similarly, NLR activation induces inflammasome assembly, activating caspase-1 and the secretion of IL-1β and IL-18, which drive Th1/Th17 differentiation 32. Adjuvants also enhance APC functions by upregulating MHC molecules, co-stimulatory markers such as CD40 and CD86, and cytokines like IL-12, promoting effective T cell priming 33. Furthermore, some adjuvants target specific pathways, such as the cGAS-STING pathway, to amplify type I IFN responses and support Th1 polarization. Beyond immediate signaling effects, adjuvants induce epigenetic modifications, such as chromatin remodeling, to sustain cytokine and co-stimulatory gene expression, establishing robust and long-lasting immune memory 34. These multifaceted mechanisms collectively enhance innate and adaptive immunity, enabling more effective vaccine responses.

### Types of adjuvants for activating CD8^+^ T cells

PRRs play a central role in the immune system by recognizing pathogen-associated molecular patterns (PAMPs) and damage-associated molecular patterns (DAMPs), thereby initiating innate immunity and shaping adaptive responses 35. In nanovaccine design, targeting PRRs with specific adjuvants enhances vaccine efficacy by enabling precise immune activation and sustained responses. This makes PRRs essential not only for guiding the categorization of adjuvants but also for tailoring immunological outcomes in vaccine development.

### TLR agonists

TLR agonists, such as monophosphoryl lipid A (MPLA, TLR4 agonist), function as adjuvants by stimulating TLRs on APCs. This activation triggers a signaling cascade that enhances the maturation and activation of these cells, leading to increased production of cytokines and co-stimulatory molecules. This, in turn, improves the presentation of antigens to T cells and boosts the overall adaptive immune response, making the vaccine more effective in eliciting a strong and long-lasting immunity. For instance, high-density lipoprotein nanodiscs co-loaded with MPLA and CpG (TLR9 agonist) significantly boost DC activation and antigen-specific CD8^+^ T cell responses. 36. In addition, lipid nanoparticles (LNPs) encapsulating type-A CpG oligodeoxynucleotides (ODNs), termed D35LNP, enhance the immunogenicity and stability of ODNs, enabling effective cancer immunotherapy. Intratumoral and intravenous administration of D35LNP in a mouse tumor model markedly suppressed tumor growth through CD8^+^ T cell activation and Th1-related gene induction 37. Importantly, a particular nano-adjuvant combining an iron oxide/gold core with a cationic polymer shell, electrostatically complexed with CpG ODNs, enhances *in situ* cancer vaccination. Integrated with irreversible electroporation for immunogenic cell death, this system shows extended tumor retention and superior tumor growth inhibition 38.

In addition to TLR4 and TLR9 agonists, bi-activation of TLR7 and TLR8 is also an effective strategy for enhancing the efficacy of immunotherapy. Notably, acidic pH-responsive poly (lactide-co-glycolide) (PLGA) nanoparticles effectively deliver the TLR7/8 agonist 522, utilizing CO_2_ gas generation for rapid cargo release at acidic sites. This inspiring formulation significantly enhances DC activation, co-stimulatory molecule expression, and MHC I-mediated antigen presentation, thereby boosting CD8^+^ T cell and NK cell responses *in vivo* 39. Additionally, TLR 7/8 bi-specific agonists encapsulated in PLGA nanoparticles significantly enhance DC activation, co-stimulatory molecule expression, and antigen presentation, improving the expansion and efficacy of antigen-specific CD8^+^ T cells 40. Interestingly, the kinetically activating-nanoadjuvant dynamically integrates t-TLR7/8a with TLR3a or TLR4a to enhance cancer immunotherapy. By coordinating dual-phase innate immune stimuli, it optimizes TLR activation and prevents immune exhaustion 41. Similarly, self-immolating nanoadjuvants co-assemble R848 and pyropheophorbide a (PPa) for targeted immunotherapy, accumulating at tumor sites and activating via acidic conditions. Laser-activated PPa induces immunogenic cell death, releasing R848 to enhance DC activation and CTL recruitment 42.

### Cytoplasmic DNA receptor activators

Cytoplasmic DNA receptor activators represent a promising class of adjuvants in nanomaterial-based immunotherapies, leveraging the cGAS-STING pathway to amplify antitumor immune responses 43. These activators function by detecting cytoplasmic DNA or delivering molecular agonists to immune cells, triggering type I interferon (IFN) production and pro-inflammatory cytokine secretion. Through the precise delivery of activators such as manganese (Mn), cyclic nucleotides, and polymeric compounds like PC7A, these systems effectively stimulate innate immunity and enhance adaptive responses, including cytotoxic T cell infiltration.

#### Mn

Mn serves as an effective adjuvant in nanomaterials for enhancing immunotherapy efficacy due to its role in activating the cGAS-STING pathway in immune cells. This activation leads to the production of type I IFN and other pro-inflammatory cytokines, which are crucial for initiating and amplifying antitumor immune responses. For instance, M@P@HA nanoparticles release Mn²⁺ and protoporphyrin in tumor cells, generating ROS under light irradiation, disrupting redox homeostasis, and releasing mitochondrial DNA to activate the STING pathway. This enhances innate immunity and promotes CD8⁺ T cell infiltration, even in low-immunogenic tumors 44. Similarly, hollow mesoporous silica-coated MnO nanoparticles are developed for MRI-guided tumor therapy, activating the cGAS-STING pathway and enhancing ROS production. These nanoparticles synergize with α-PD-1 therapy, promoting CTL infiltration and effectively curtailing melanoma progression and metastasis 45.

#### Cyclic nucleotides

Cyclic nucleotides encapsulated in nanomaterials target the STING pathway, triggering a strong immune response through controlled release. This approach ensures localized activation of immune cells, boosting type I IFN and cytokine production for effective antitumor immunity. For instance, a distinct lipid-based delivery system comprising neutral cytidinyl lipid DNCA and cationic lipid CLD enhances the delivery and efficacy of cyclic dinucleotides in cancer immunotherapy. This system potently stimulates IFN production, promotes immunogenic cell death, and activates NK and CD8^+^ T cells, significantly inhibiting tumor growth 46. Additionally, mesoporous silica nanoparticles, modified with poly (ethylene glycol) and a cationic molecule, successfully deliver cyclic diguanylate monophosphate into the TME. This nanoplatform activates the STING pathway in APCs, significantly enhancing leukocyte infiltration and cytokine secretion, thereby inhibiting tumor growth in breast cancer models 47.

#### PC7A

PC7A functions as an adjuvant by activating the STING pathway, promoting robust immune responses. Its polymeric structure facilitates efficient antigen delivery to immune cells and enhances their uptake, while its gradual degradation ensures sustained activation. This prolonged stimulation of the STING pathway drives type I IFN production and antigen presentation, enhancing the efficacy of immunotherapies and improving treatment outcomes. Intratumoral administration of a STING-activating PC7A nanovaccine enhances antitumor efficacy compared to subcutaneous delivery by significantly improving the infiltration of antigen-specific cytotoxic T cells into tumors. This effect is mediated through a positive feedback loop involving CXCL9 expression by myeloid cells and IFN-γ by T cells 48. Additionally, combining local radiotherapy with a systemic PC7A STING-activating nanovaccine significantly enhances antitumor immunity and leads to regression of advanced solid tumors in mouse models 49.

### Cytoplasmic RNA receptor activators

**Figure 3:**
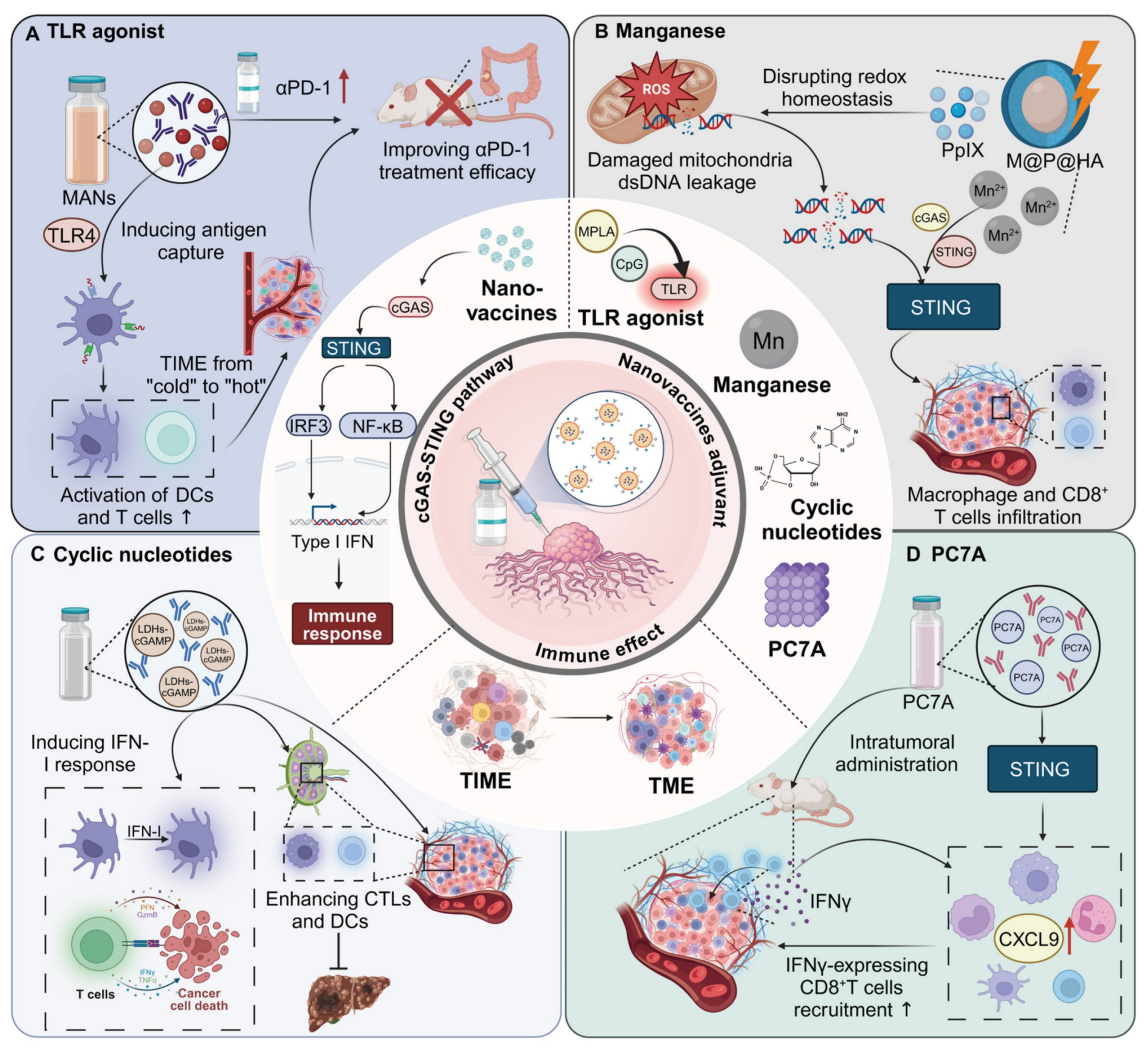
** Overview of types and mechanism of nanovaccine adjuvants.** (A) Nanovaccines loaded with TLR4 agonists induce DC maturation and antigen capture, transforming the TME from an immunologically “cold” to “hot” state. This leads to increased activation of DCs and T cells, thereby improving the efficacy of αPD-1 therapy in colorectal cancer models. (B) Manganese-loaded nanovaccines, via oxidative stress and mitochondrial DNA leakage, disrupt cellular redox homeostasis. This leads to the activation of the cGAS-STING pathway, inducing type I IFN responses and facilitating CD8^+^ T cell infiltration and macrophage activation within the tumor, thus promoting a robust anti-tumor immune response. (C) Nanovaccines loaded with cyclic nucleotides stimulate the cGAMP-STING signaling pathway, resulting in enhanced CTL and DC responses in both tumor and lymphoid tissues. This approach significantly enhances type I IFN signaling and inhibits hepatocellular carcinoma growth. (D) Intratumoral administration of PC7A-loaded nanovaccines stimulates CXCL9 expression in myeloid cells, leading to increased recruitment of IFNγ-expressing CD8^+^ T cells into the TME. This upregulation of CXCL9 enhances anti-tumor immune responses through efficient CD8^+^ T cell activation and infiltration into the tumor.

Another critical PRR expressed in APCs is the cytoplasmic RNA receptor family, RLRs, which includes RIG-I and MDA5Agonists of these receptors, such as 5'ppp-dsRNA, poly(I:C), and poly(dA:dT), represent a promising class of adjuvants in cancer vaccines. These molecules activate innate immune responses through type I IFN induction and pro-inflammatory signaling, transforming immunosuppressive tumor TME into immunogenic ones. For instance, the lipid calcium phosphate nanoparticle platform, capable of co-encapsulating phosphorylated tumor-specific peptides and adjuvants, demonstrated potent anti-cancer efficacy. Among tested formulations, the RIG-I agonist 5'pppdsRNA showed superior performance in reducing primary colon cancer growth and preventing liver metastasis compared to TLR9 (CpG) and STING (2'3'cGAMP) adjuvants. 5'pppdsRNA enhanced CD8^+^ T-cell responses while avoiding immunosuppressive cell expansion, transforming the TME into a more immunogenic state. This underscores its potential as a cancer vaccine adjuvant, particularly in combination therapies targeting the suppressive TME 50. Interestingly, poly(I:C) acts as an adjuvant to enhance CAR-T cell therapy by promoting IL-2 and IFN-γ production and boosting cytolytic activity. Systemic administration of poly(I:C) significantly suppresses tumor growth, reduces MDSC numbers and attenuates their immunosuppressive activity 51. Moreover, poly(dA:dT), delivered via DEC205-specific antibodies, acts as an adjuvant by inducing type I IFN in DCs. Combined with a DC-targeted antigen, it elicits adaptive T cell immunity, demonstrating the efficacy of dual delivery to DCs in vaccination strategies 52** (Figure 3) (Table 1)**.

## Types of nanovaccine for activating CD8^+^ T cells

Nanovaccines for activating CD8^+^ T cells can be categorized based on their loaded components into nucleic acid-based nanovaccines and antigen-based nanovaccines. Nucleic acid-based nanovaccines and antigen-based nanovaccines both function by leveraging tumor antigens to elicit immune responses, although their delivery mechanisms differ 53. In the field of cancer immunotherapy, selecting between nucleic acid-based and antigen-based nanovaccines is largely influenced by the TME and the specific requirements of the immune response. Nucleic acid vaccines, encompassing DNA and RNA technologies, excel at exploiting the host's cellular mechanisms to produce tumor-specific antigens internally. This approach mimics a natural immune response against tumor cells by engaging both MHC class I and II pathways, effectively activating a broad range of immune cells, including both cytotoxic and helper T cells 54, 55. This capability is crucial in cancer therapy, where the immune system must adapt to the evolving antigenic profile of tumors, potentially addressing multiple tumor variants at once. However, these vaccines face significant challenges related to their delivery and stability. DNA vaccines need to reach the nucleus, which can be inefficient in the non-dividing cells that are typical in many tumors. RNA vaccines, while capable of rapid antigen translation, are inherently unstable and require protection in complex delivery systems, such as LNPs, to prevent degradation and ensure effective delivery to target cells. These systems often need stringent cold chain management, which can be logistically challenging 56, 57. Conversely, antigen-based vaccines can precisely target known tumor antigens and are typically easier to store and handle, bypassing the extensive cold chain requirements needed for many nucleic acid-based vaccines 18. However, the static nature of the antigens in these vaccines can limit their effectiveness, particularly as tumors evolve and present new or altered antigens that are not recognized by the immune system initially primed by the vaccine. Additionally, the immune responses triggered by these vaccines are often short-lived, which may necessitate repeated doses to sustain therapeutic efficacy, posing a significant challenge in ongoing cancer management 58.

### Antigen-based nanovaccine for enhancing the efficacy of CD8^+^ T cell-based immunotherapy

Antigen-based vaccines represent a cornerstone of cancer immunotherapy, owing to their ability to activate CD8^+^ T cells and elicit potent anti-tumor immune responses. Recent technological advancements have spurred the development of inspiring vaccine platforms, each offering unique advantages in antigen delivery efficiency, stability, and immune activation. The incorporation of nanotechnology has revolutionized antigen-based vaccine strategies by enabling precise and efficient antigen delivery through nanoparticle carriers, thereby amplifying CD8^+^ T cell activation. Simultaneously, nucleic acid vaccines have emerged as a cutting-edge approach, harnessing the host's cellular machinery to produce and present antigens *in situ*, inducing robust and durable immune responses. These approaches not only exhibit distinct advantages in enhancing CD8^+^ T cell-mediated immunity but also demonstrate complementary mechanisms of action, underscoring their potential for synergistic applications. Collectively, these advancements highlight the transformative potential of antigen-based vaccines in cancer immunotherapy and warrant further exploration into their clinical utility.

### Nanovaccines: direct delivery of antigens for robust CD8^+^ T cell activation

#### Tumor-derived antigen

**Figure 4:**
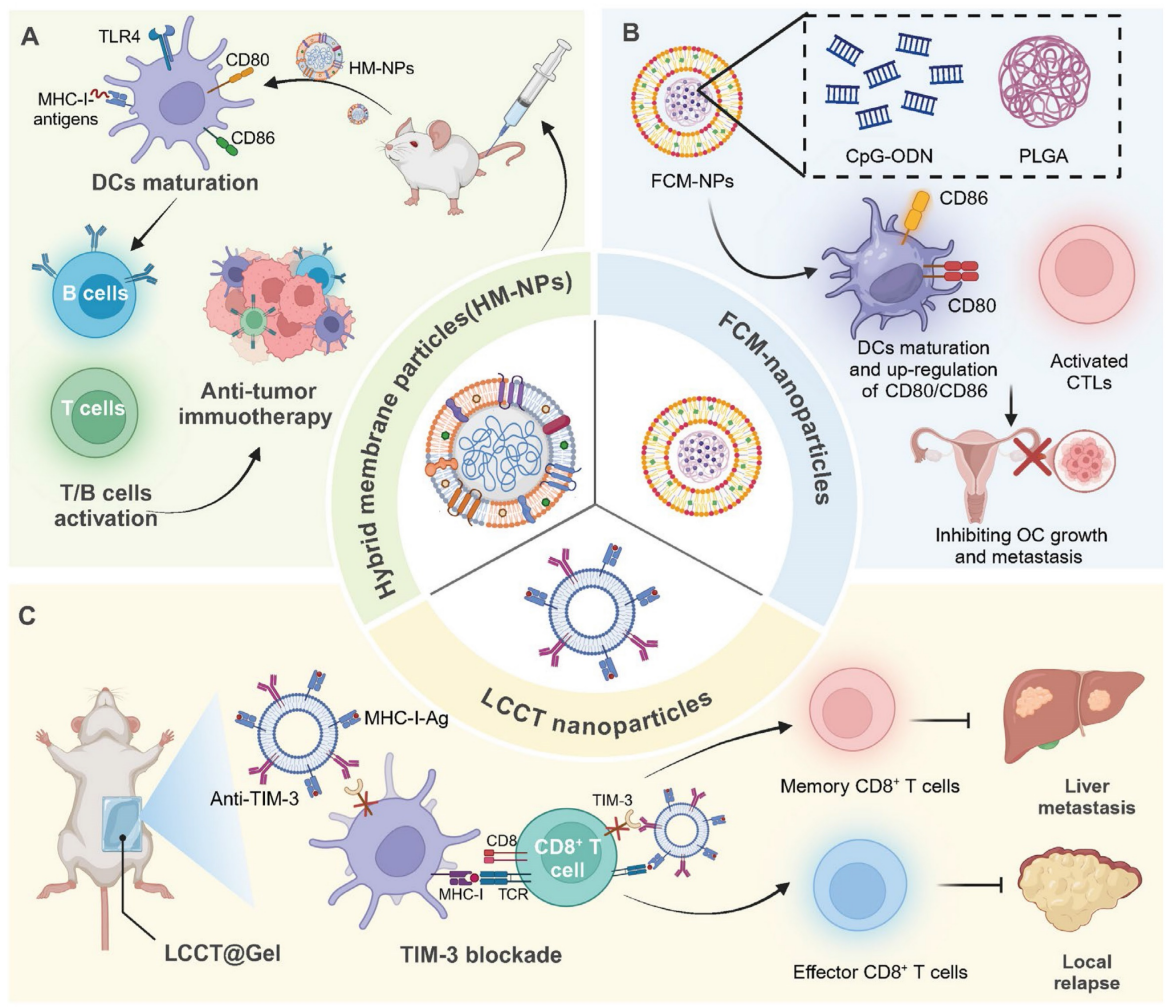
** Tumor cell membrane-based nanovaccines: advancing cancer immunotherapy.** (A) HM-NPs stimulate DC maturation via TLR4 activation, enhancing CD80/CD86 expression and antigen presentation, which subsequently activates T and B cells, leading to a potent anti-tumor immune response. (B) Ovarian cancer poses significant health risks to women. To improve immunotherapy, FCM-NPs were developed by fusing DC and OC cell membranes onto CpG-ODN-loaded PLGA nanoparticles. FCM-NPs enhanced DC maturation, lymph node homing, and tumor antigen presentation, effectively activating tumor-specific CD8^+^ T cells. It demonstrated strong antitumor effects, inhibiting OC growth and metastasis, highlighting its therapeutic promise. (C) LLCT nanoparticles combined with TIM-3 blockade facilitate antigen presentation by DCs to CD8^+^ T cells, promoting the generation of both memory and effector T cells, effectively controlling liver metastasis and preventing local relapse.

Incorporating tumor-derived antigens into nanomaterials enables a more precise targeting and activation of the immune system against cancer cells. For instance, a hybrid membrane cancer vaccine, incorporating ginseng-derived extracellular vesicle-like particles with membranes from resected tumors, forms a codelivery system that enhances DC phagocytosis and maturation via TLR4 activation. This vaccine efficiently stimulates tumor-specific CTLs, offering robust immune responses that reduce tumor recurrence and metastasis, while also extending survival in animal models 59. Similarly, DC-derived nanovesicles conjugated with αCTLA-4 and presenting tumor antigens selectively activate tumor-specific CTLs. This nanoplatform significantly enhances the antitumor efficacy of αCTLA-4 therapy while reducing immune-related adverse events in syngeneic tumor models 60. In addition, glycoliposomes modified with the glycan Lewis X specifically target DC-SIGN receptors on DCs, effectively delivering tumor antigens and MPLA for enhanced DC activation and pro-inflammatory cytokine production. This targeted delivery system significantly improves the cross-presentation of the melanoma antigen gp100 to CD8^+^ T cells and outperforms non-glycosylated liposome formulations 61. Notably, magnetic liposomes containing melanoma antigens and iron oxide nanoparticles, enhanced by CpG-1826, target lymph nodes for effective antigen presentation. This targeting promotes DC activation and CTL responses, significantly inhibiting tumor growth 62 **(Figure 4)**.

In addition to nanovesicles and liposomes, polymer-based delivery platforms have also been developed for delivering tumor derived antigens. For instance, a personalized cancer vaccine, using poly (lactic-co-glycolic acid) nanoparticles functionalized with polyethylenimine and coated with polyinosinic:polycytidylic acid, encapsulates patient-derived melanoma neoantigens to effectively stimulate DCs and lymphocytes. This nanovaccine demonstrates superior immune activation, efficiently maturing DCs for antigen presentation and enhancing lymphocyte cytotoxicity, thereby providing a targeted and potent response against melanoma cells 63. Similarly, polyethyleneimine -based nanoparticles offer a streamlined approach for personalized cancer vaccine production, utilizing simple modular programming for neoantigen and CpG adjuvant integration. These sub-50 nm nanoparticles effectively activate APCs and neoantigen-specific CD8^+^ T cells, inducing strong antitumor effects against local and metastatic tumors with a single intratumoral injection 64. In addition, a PLGA-based nanoparticle vaccine incorporating the cancer germline antigen NY-ESO-1 and the iNKT cell agonist IMM60 has been developed to activate DCs and enhance T cell responses. This nanovaccine efficiently presents peptides across various HLA subtypes, significantly boosting both CD4^+^ and CD8^+^ T cell activity and antibody production against NY-ESO-1 65. Likewise, a fusion cell membrane (FCM) nano-vaccine, encapsulating dendritic and ovarian cancer cells in PLGA nanoparticles with CpG-ODN, enhances DC maturation and T-cell activation. Demonstrating potent immunogenicity and antigen-presenting capabilities, FCM-nanoparticles effectively delay tumor growth and inhibit metastasis in ovarian cancer models 66.

Notably, inorganic nanomaterials have also been utilized for antigen delivery. For instance, spike-topology silicon nanoparticles efficiently co-deliver hepatocellular carcinoma-specific neoantigens and TLR9 agonists to DCs, enhancing CD8^+^ T cell and central memory T cell responses. This approach supports robust anti-tumor effects in orthotopic liver cancer models and, combined with TIM-3 blockade, shifts the TME, decreasing Tregs and metastasis 67. In addition, incorporating TAAs and CpG into calcium-based nanosystems effectively activates immune responses. The pH-sensitive CaCO_3_ nanovaccine loaded with tumor cell lysates and CpG adjuvant neutralizes the acidic TME, enhances M1 macrophage proportion, and promotes effector cell infiltration 68. Likewise, calcium phosphate nanoparticles, functionalized with CpG and tumor antigens, boost cytotoxic CD8^+^ T cell responses in a murine colorectal cancer model, enhancing specific immunity and tumor suppression 69. MnO_2_-melittin nanoparticles have been engineered to effectively harness the TME, catalyzing a Fenton-like reaction to induce tumor cell death and stimulate the cGAS-STING pathway. These nanoparticles enhance antigen presentation, boost tumor-specific T cell responses, and increase pro-inflammatory signals, outperforming individual components in reducing tumor growth and metastasis 70.

In recent years, various nanodelivery platforms have been developed to enhance antigen delivery and immune activation. Interestingly, a plant virus nanoparticle, specifically the cowpea mosaic virus, has been engineered to display NY-ESO-1 antigens, enhancing APC activation and stimulating robust CD8^+^ T cell responses. This bio-inspired nanomaterial platform induces potent antitumor immunity in transgenic mice expressing human HLA-A2, demonstrating its potential for targeting NY-ESO-1^+^ malignancies such as breast cancer and melanoma 71. In addition, the LCCT nanovaccine, integrating a lncRNA-edited tumor cell membrane with anti-TIM-3, enhances DC activation and T cell responses in TME. Embedded in an alginate-based hydrogel, it effectively prevents post-surgical tumor relapse by boosting antigen cross-presentation and reducing T cell immunosuppression 72.

#### Synthetic antigen

By embedding synthetic peptides into nanoparticles, immunotherapies can effectively introduce different epitopes to the immune system. These designed peptides can be tailored to enhance immune recognition, providing a versatile platform for eliciting strong and specific immune responses, which are essential for overcoming the diverse and adaptive nature of cancer cells. For instance, the cationic lipid R-DOTAP, when formulated with antigenic peptides, stimulates robust antitumor CD8^+^ T cell responses through a Myd88-dependent, type I IFN-mediated pathway involving endosomal TLR7/9 activation. This lipid formulation induces complete tumor regression in a human papillomavirus model and enhances the efficacy of PD-1 checkpoint inhibitors in melanoma 73. Similarly, a nano-liposomal vaccine integrating P5 peptide, PADRE peptide, and MPLA enhances CTL and T-helper responses, significantly boosting anti-tumor immunity against HER2^+^ cells in BALB/c mice. This formulation, by co-activating CD8^+^ and CD4^+^ T cells and stimulating IFN-γ production, demonstrates improved survival and potent antitumor effects 74.

Various polymer- and inorganic material-based nanodelivery platforms have been developed for the efficient delivery of synthetic antigens. A polymeric nanovaccine platform incorporating a TLR7/8 agonist and an endosomal escape peptide activates the NLRP3 inflammasome, enhancing neoantigen delivery and immunogenicity. This platform induces strong CD8^+^ T cell responses and, when combined with checkpoint blockade, effectively combats established tumors in multiple cancer models 75. Similarly, the nanovaccine 8FNs, combining a peptide-based tumor antigen FK-33 with a poly (ester amide) delivery system, activates DCs and elicits a potent CD8^+^ T cell response. 8FNs significantly inhibit tumor growth and metastasis and synergize with PD-1 therapy, demonstrating their potential for personalized cancer immunotherapy 76. In addition, a specific polyethyleneimine-based nanoparticle vaccine, encapsulating neoantigen peptides and CpG adjuvants, enhances DC activation and CD8^+^ T cell priming. Despite limited initial tumor infiltration, subsequent local administration of a STING agonist significantly improves infiltration and anti-tumor efficacy 77. Moreover, PLGA nanoparticles encapsulating a heteroclitic BCMA72-80 peptide have been developed to target multiple myeloma, exhibiting enhanced delivery and CTL induction capabilities compared to liposomal formulations. These nanoparticles facilitate sustained antigen presentation, resulting in robust BCMA-specific CTL responses characterized by increased memory CTL populations, cytokine production, and anti-tumor activities 78. Engineered aluminum nanoparticles delivering dual-epitope peptides enhance T cell activation in cancer immunotherapy. These nanoparticles are modified with a PEG derivative to stabilize and optimize antigen delivery to APCs. Enhanced uptake by these cells results in improved CD8^+^ T cell response and significant tumor suppression in mouse models 79. Notably, manganese-doped silica nanoparticles (Mn^4+^-SNPs) combined with the HPV16 E7-derived peptide GF001 create a potent therapeutic HPV vaccine. Mn^4+^-SNPs function as self-adjuvants, enhancing inflammatory pathways and immune cell recruitment, and facilitate antigen delivery for effective cross-presentation. This nanovaccine induces robust CD8^+^ T cell responses, achieving remission in murine models of HPV-related tumors 80. Likewise, mesoporous silica nanoparticles are engineered to target DCs and enhance T-cell activation, utilizing a peptide TY, OVA and CpG. This targeted nanovaccine effectively promotes DC maturation and antigen-specific CD8^+^ T cell activation *in vitro* and *in vivo*, demonstrating significant antitumor effects and improved survival in tumor-bearing mice with minimal systemic toxicity 81.

Self-assembling nanovaccines have emerged as an inspiring and promising approach for the effective delivery of synthetic antigens. The SNP-7/8a vaccine platform utilizes charge-modified peptide-TLR-7/8a conjugates to self-assemble into uniform nanoparticles, effectively loading and delivering diverse peptide neoantigens alongside adjuvants. This enhances the uptake and activation of APCs, boosting CD8^+^ T cell immunity and tumor clearance in mice and primates 82. Additionally, twelve self-assembling conjugates combining MAGE-A1 peptide with a TLR2 agonist were synthesized, forming nanoparticles that enhance peptide vaccine efficacy through improved lymph node targeting and plasma stability. Conjugate 6 notably advanced DC maturation, CD8^+^ T cell activation, and demonstrated a 70% tumor inhibition rate 83. Notably, the TLR7/8 agonist-epitope conjugate forms a self-assembling, carrier-free nanovaccine that enhances DC activation via MyD88-dependent TLR signaling. This nanoformulation prolongs retention, improves lymphatic drainage, and markedly boosts CD8^+^ T cell immunity. Demonstrating significant therapeutic effects against melanoma in mice, this nanovaccine offers a streamlined approach for personalized cancer immunotherapy 84.

#### Model antigen

Utilizing OVA within nanomaterials as a model antigen provides a controlled and reproducible framework for dissecting immune mechanisms. This experimental approach facilitates detailed investigations into antigen processing and presentation dynamics, thereby enhancing the understanding of immune system interactions with nanocarriers. Various nanodelivery platforms based on polymer materials have been developed for the efficient delivery of OVA. For instance, chitosan nanoparticles (CNPs) effectively deliver antigens, enhancing DC activation and CD8⁺ T cell expansion. CNPs loaded with the SIINFEKL peptide from OVA induce potent immune responses, enabling tumor cell lysis in a pancreatic cancer model 85. Similarly, targeting DC receptors with pegylated PLGA nanoparticles encapsulating OVA and TLR ligands enhances cytotoxic T cell responses. Specific monoclonal antibodies directed at DC surface molecules CD40, DEC-205, and CD11c improved nanoparticle internalization, IL-12 production, and T cell activation 86. In addition, PLGA nanoparticles encapsulating the OVA antigen significantly enhance T cell activation and cross-presentation capabilities *in vitro*. When primed with these nanoparticles, the Gr-1 positive polymorphonuclear cells from mouse bone marrow effectively induce OT-I CD8^+^ T cell proliferation and alter cell division and morphology 87. Interestingly, a subunit vaccine platform uses *in situ* polymerization of C7A and acrylamide to encapsulate OVA, significantly enhancing CD8^+^ T cell activation via improved MHC-I-mediated antigen presentation. This nanotechnology-driven approach also boosts B cell activation, antibody and cytokine production, and demonstrates potent immune memory formation with a favorable safety profile 88.

Inorganic material-based nanodelivery systems have also been used for delivering OVA efficiently. Redox-responsive OVA nanoparticles, assembled via zeolitic imidazolate framework-8 and conjugated with TLR7/8 agonists, enhance lymph node-targeted immune activation. These nanovaccines facilitate potent DC activation and antigen cross-presentation, triggering robust CTL responses and durable immunological memory 89. Similarly, magnetic nanoparticles covalently modified with OVA254-267 antigen and CpG oligonucleotide enhance DC activation and sustain CD8^+^ T cell responses against melanoma more effectively than free components. These nanoparticles demonstrate stability in blood and potentiate systemic, tumor-specific CD8^+^ T cell activation, showcasing their efficacy in immunotherapy 90. Notably, polyelectrolyte multilayer (PEM) coatings on gold nanoparticles, termed immune-PEMs (iPEMs), efficiently deliver OVA peptide and TLR agonists to DCs, enhancing T cell activation and antigen presentation. These iPEMs, assembled through electrostatic interactions without solvents, allow precise control of immune signal composition and demonstrate superior antigen-specific CD8^+^ T cell activation 91. In addition, iron oxide-embedded large-pore mesoporous organosilica nanospheres have been developed to activate cytotoxic T cells and repolarize TAMs from an M2 to an M1 phenotype. These nanospheres, loaded with the antigen OVA, effectively enhance DC-mediated T cell responses and induce TAM-mediated tumor cell apoptosis 92.

Building on these advancements, different nanovaccine delivery systems have been developed to optimize OVA delivery and enhance immune responses. For instance, protein cage nanoparticles, encapsulin (Encap), genetically modified with the OT-1 peptide from OVA, serve as efficient antigen carriers for cancer immunotherapy. They enhance antigen delivery to DCs, triggering CD8^+^ T cell activation and robust cytotoxic responses. In murine melanoma models, OT-1-encap vaccinations significantly suppressed tumor growth, demonstrating their potential for targeted epitope-specific T cell activation 93. Moreover, virus-like particles (VLPs) derived from phage P22, engineered to display OVA-derived epitopes (VLP-OVAB and VLP-OVAT), effectively deliver peptide antigens for cancer immunotherapy. VLP-OVAT notably enhances CTL responses and inhibits tumor growth by modulating T cell populations and reducing immunosuppressive cells in tumor models 94. Interestingly, an intranasal self-assembled nanovaccine (I-OVA NE) efficiently delivers epitope peptides (IKVAV-OVA257-264) for tumor immunotherapy. Exhibiting optimal particle size and stability, I-OVA NE significantly increases nasal retention and integrin-mediated uptake. This nanovaccine substantially enhances CD8^+^ T cell activation and Th1 immune responses, demonstrating effective protective immunity against E.G7-OVA tumors 95** (Table 2).**

### Nucleic acid vaccines: endogenous antigen expression for enhanced immune responses

**Figure 5:**
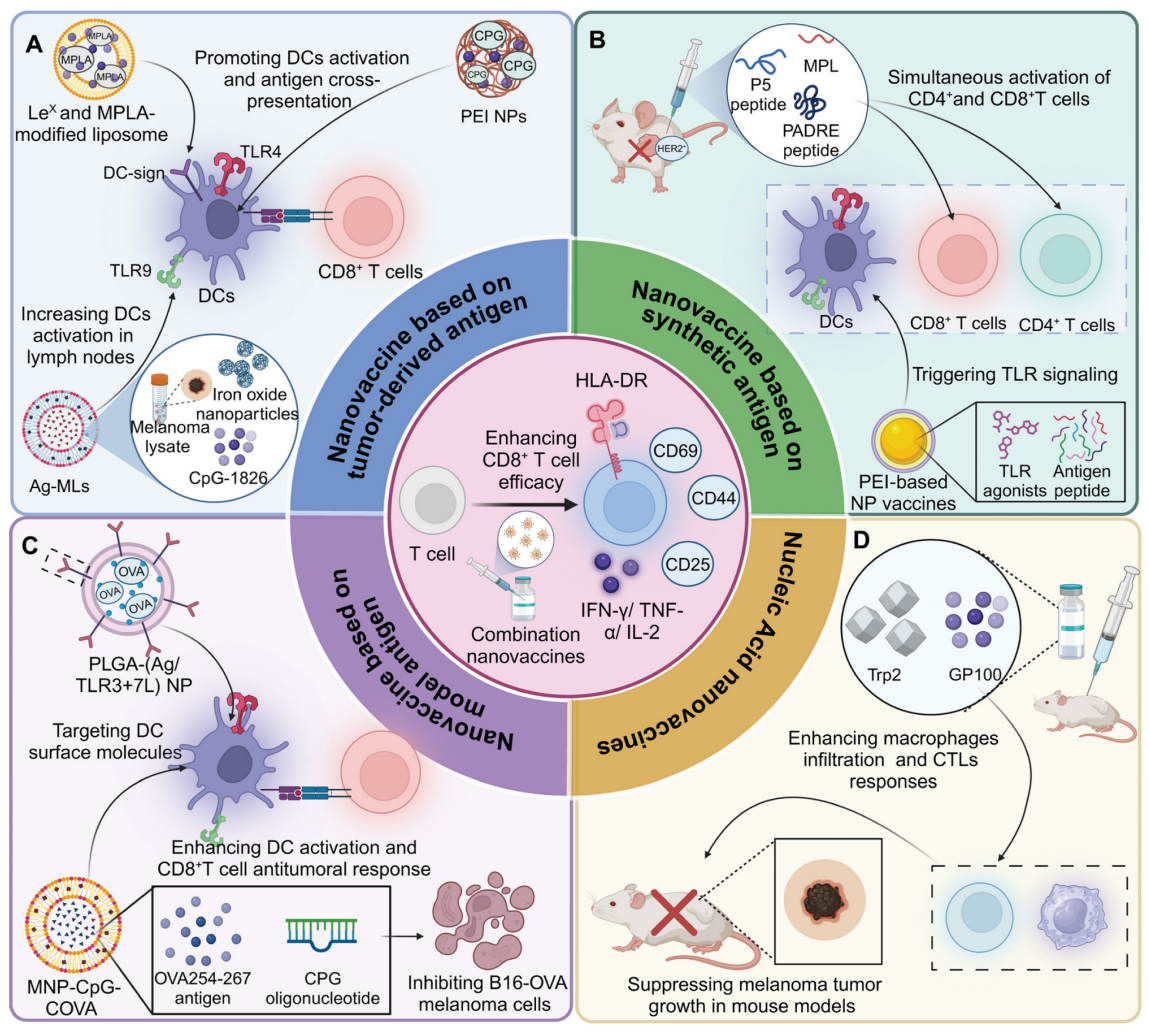
** Antigen-based nanovaccines and nucleic acid nanovaccines for enhancing CD8^+^ T cell-mediated anti-tumor immunity.** (A) Lex-modified and MPLA-liposomes and Ag-MLs are taken up by DCs via the DC-SIGN receptor, promoting DC activation and cross-presentation of tumor antigens, which increases the population of active DCs in lymph nodes and triggers CD8^+^ T cell responses. (B) P5-PADRE-MPLA nano-liposomal vaccines and PEI-based NPs significantly boost antitumor immunity by enhancing CTL and T-helper responses. (C) Targeted delivery of antigens using PLGA nanoparticles decorated with TLR3 and TLR7 agonists efficiently activates DCs by targeting surface molecules. This results in enhanced CD8^+^ T cell-mediated anti-tumor responses, effectively inhibiting melanoma cell growth. MNP-CpG-COVA nanovaccines facilitate MHC-I antigen presentation and robust CD8^+^ T cell responses by promoting neoantigen presentation on DCs. DNA origami containing CpG ODN further enhances the immunogenicity of neoantigens in this context, resulting in the inhibition of B16-OVA melanoma cells. (D) DLNA vaccines carrying the melanoma epitopes Gp100 and Trp2 induce increased macrophage infiltration and CTL responses, resulting in CD8^+^ T cell-dependent suppression of melanoma growth.

When encapsulated in nanoparticles, DNA can be efficiently delivered into cells, where it directs the synthesis of antigens that elicit a targeted immune response. This method leverages the body's natural cellular processes to stimulate immunity, making DNA-based nanovaccines a powerful tool for developing long-lasting and adaptive immune responses against various diseases. For instance, an inspiring RGD-based nanoparticle vaccine, encapsulating an E749-57-HSP110 fusion expression plasmid, effectively targets and penetrates TC-1 tumor cells, stimulating robust CD8^+^ T cell activation and IFN-γ production. This targeted delivery and enhanced immunogenicity result in significant tumor suppression and extended survival in cervical cancer mouse models. 96. Similarly, nanogels (Alg-Tat-gp100) enhance oral plasmid DNA vaccine delivery by improving stability and intestinal penetration. In mice, they significantly boost IFN-γ secretion and cytotoxic T cell activation, achieving significant tumor inhibition in melanoma 97. Interestingly, electroporation-facilitated, DNA-launched nanoparticle vaccines (DLnano-vaccines) enhance CD8^+^ T cell responses and tumor control. DLnano-vaccines with melanoma epitopes Gp100 and Trp2 elicit stronger CTL responses than DNA monomeric or CpG-adjuvanted peptide vaccines, suppressing melanoma growth in a CD8^+^ T cell-dependent manner 98 **(Figure 5)**.

#### mRNA-based nanovaccines

**Figure 6:**
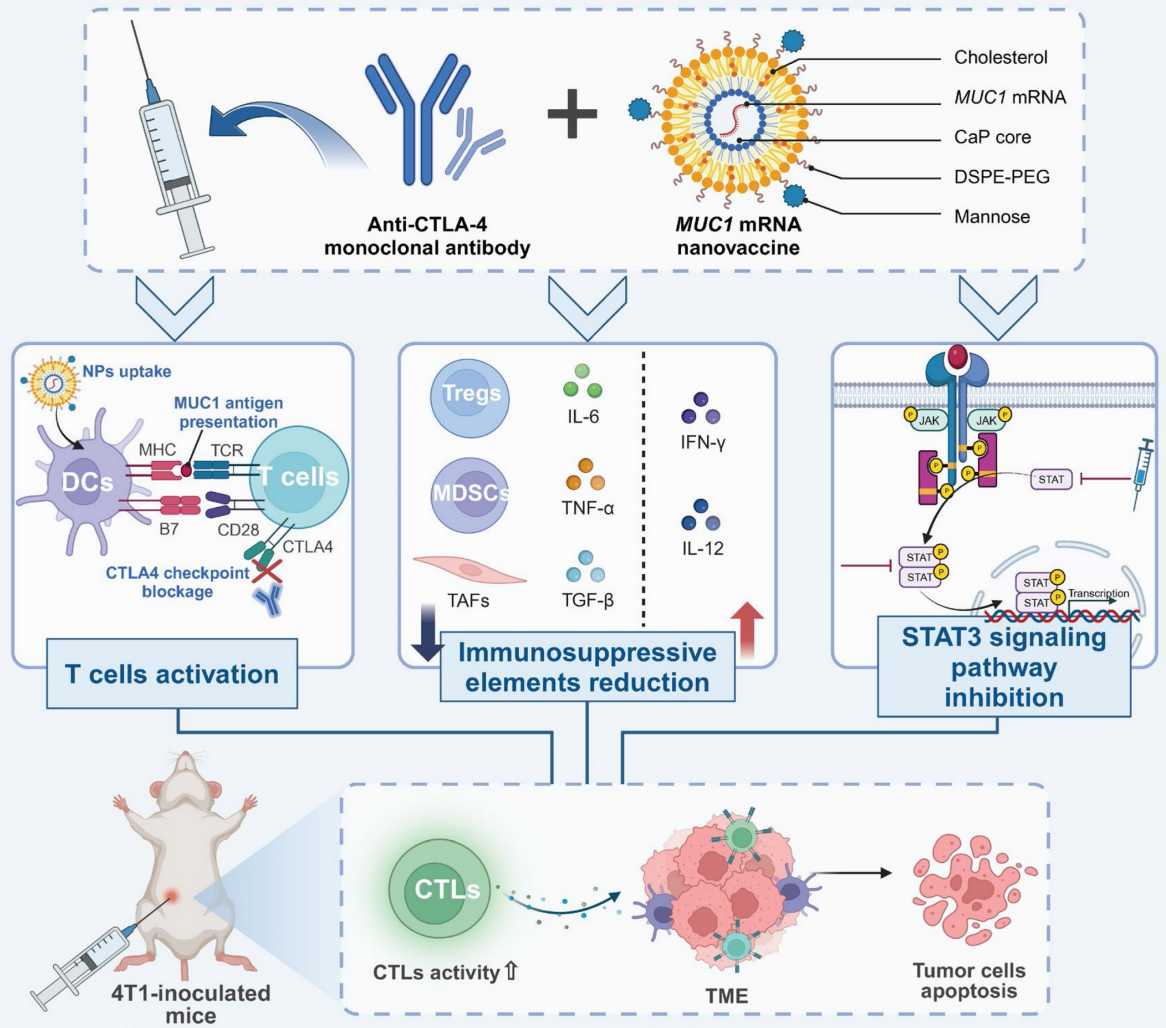
** Enhanced CTL responses in TNBC via *MUC1* mRNA nanovaccine and anti-CTLA-4 therapy.** Combined immunotherapy with anti-CTLA-4 antibodies and *MUC1* mRNA nanovaccines enhances anti-tumor immunity by promoting T cell activation and reducing immunosuppression in the TME. MUC1 nanovaccines are taken up by dendritic cells, leading to antigen presentation and robust CTL activation, amplified by CTLA-4 blockade. This increases CTL infiltration into tumors, triggering apoptosis. The therapy also reduces immunosuppressive cells such as Tregs and myeloid-derived suppressor cells, while upregulating pro-inflammatory cytokines like IFNγ. Additionally, STAT3 pathway inhibition suppresses tumor-supportive signals, further driving tumor cell death. In a 4T1 mouse model, this combined approach results in significant CTL-mediated tumor regression.

mRNA is crucial for nanovaccines because it serves as a transient template that instructs host cells to produce specific antigens, thereby inducing a targeted immune response. Encapsulated within nanoparticles, mRNA ensures the safe delivery and translation of genetic information directly into the cellular machinery, bypassing the need for DNA integration and accelerating the speed at which immunity can be achieved. Combining *MUC1* mRNA nanovaccine with anti-CTLA-4 monoclonal antibody enhances CTL responses in TNBC by reducing immunosuppressive elements within the TME. This synergy decreases regulatory T cells and myeloid-derived suppressor cells, downregulates pro-tumorigenic cytokines and STAT3 signaling, and increases tumor cell apoptosis, providing a potentiated immunotherapeutic strategy 99. Similarly, nanoparticles designed to deliver an mRNA vaccine targeting the MUC1 tumor antigen to DCs via mannose receptors have demonstrated significant efficacy in activating CTL against TNBC *in vivo* 100 **(Figure 6)**. In addition, a particular lipid nanoparticle formulation enhances the delivery of mRNA vaccines, effectively inducing cytotoxic CD8^+^ T cell responses. Demonstrated in a B16F10 melanoma model, this approach shows potent activation of T cells and significant tumor shrinkage post-immunization. The inclusion of the adjuvant LPS further amplifies immune responses, underscoring the formulation's potential as a powerful vector for mRNA vaccine delivery in cancer immunotherapy 101.

The selection and deployment of antigens and their delivery systems are critical to achieving targeted and effective treatment outcomes. Tumor-derived antigens, extracted directly from an individual's tumor cells, offer a highly personalized approach by harnessing the unique antigenic profile of the tumor. This method ensures that the immune response is specifically tailored to the individual's cancer, potentially increasing the precision and efficacy of the therapy 102. However, the variable nature of these antigens and the complex, costly production process limit their widespread application. In contrast, synthetic antigens provide a more scalable option, allowing for the precise targeting of known tumor-associated epitopes and mass production capabilities. Yet, they might not cover the entire spectrum of tumor mutations and risk immune tolerance if they mimic self-antigens too closely. Model antigens, such as OVA, are invaluable in research settings for dissecting immune mechanisms and optimizing vaccine formulations but fall short in clinical applicability due to their oversimplified representation of human TME.

Notably, a lipid nanoparticle-encapsulated mRNA vaccine encoding HPV E7 protein activates CD8^+^ T cells and modulates their functional commitment in both the spleen and TME. When combined with immune checkpoint inhibitors, this vaccine enhances HPV-specific CD8^+^ T cell responses and promotes tumor regression in HPV-positive oropharyngeal squamous cell carcinoma 103.

**Figure 7:**
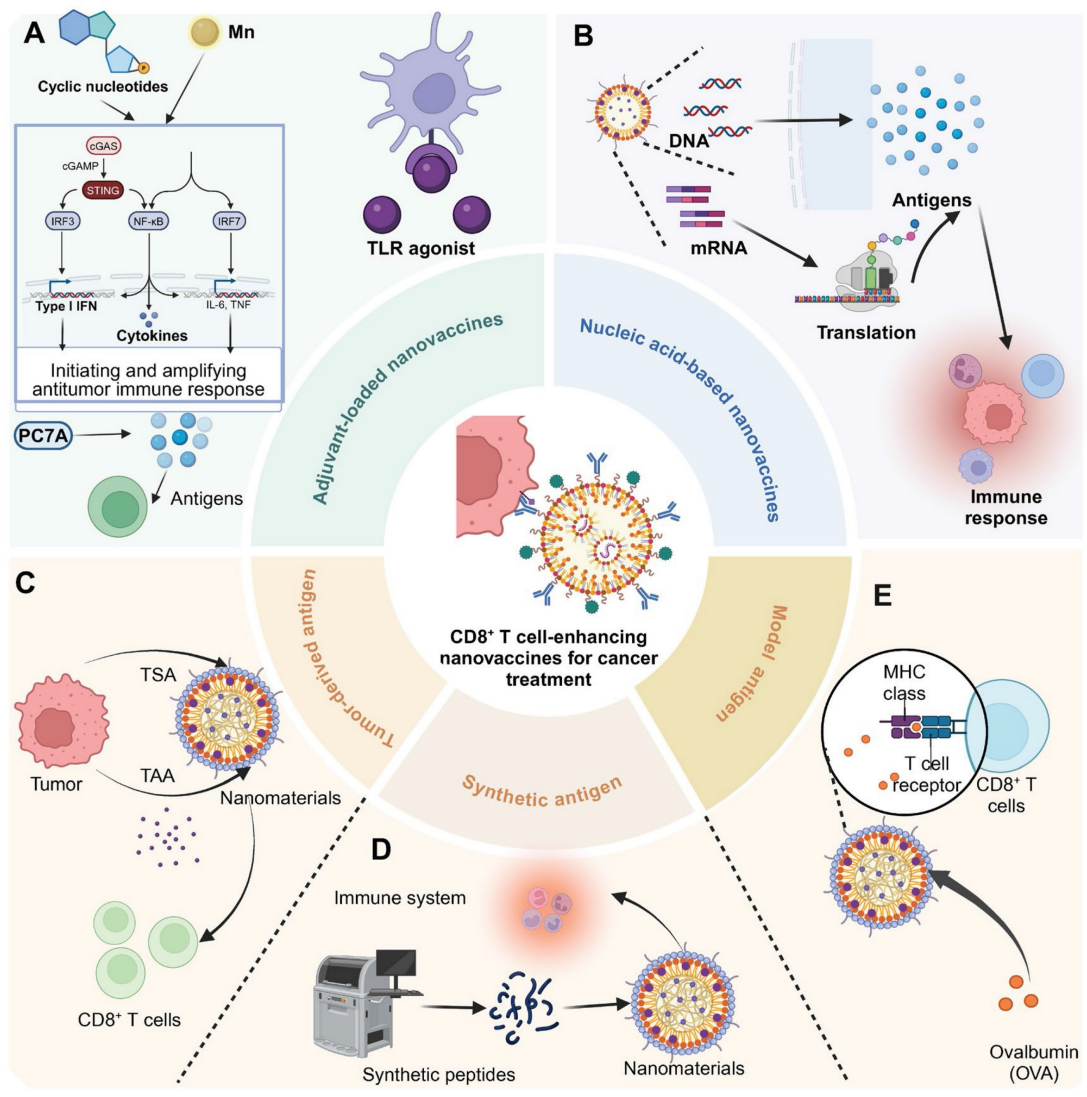
** CD8^+^ T cell-enhancing nanovaccines for cancer treatment.** (A) In adjuvant-loaded nanovaccines, cyclic nucleotides and Mn2^+^ activate the cGAS/STING pathway, leading to the production of type I IFN and inflammatory cytokines (IL-6, TNF) via IRF3/NF-κB/IRF7 signaling cascade. PC7A polymer facilitates antigen delivery and immune activation. TLR agonist further enhances immune responses through APC activation. (B) Nucleic acid-based nanovaccines carry DNA or mRNA cargo. After cellular uptake, these genetic materials undergo translation to generate antigens that trigger immune responses. (C) Tumor-derived antigen strategy utilizes tumor-specific antigens (TSA) and tumor-associated antigens (TAA) from tumor cells, which are incorporated into nanomaterials to stimulate CD8^+^ T cell responses. (D) Synthetic peptide approach employs artificially synthesized peptides that are formulated into nanomaterials. (E) Model antigen ovalbumin (OVA) demonstrates the interaction between nanovaccine-delivered antigens and CD8^+^ T cells through MHC class I presentation and T cell receptor recognition. These complementary strategies aim to enhance antitumor immune responses through CD8^+^ T cell activation.

Additionally, three mRNA vaccine modalities, encapsulated in lipid nanoparticles, were tested against HPV-16 induced tumors in mice, demonstrating robust activation of E7-specific CD8^+^ T cells and memory responses. These vaccines eradicated established tumors and offered potent protection in orthotopic models, showing superior efficacy compared to DNA and protein vaccines, supporting their advancement to clinical trials 104 **(Table 3) (Figure 7)**.

## Comparative evaluation of nanodelivery systems for antigen delivery in cancer immunotherapy

The selection of nanodelivery systems for antigen delivery in cancer immunotherapy necessitates a rigorous assessment of their specific advantages and limitations to ensure efficient, targeted, and safe immune activation. Nanovesicles, derived from cell membranes, exhibit excellent biocompatibility and leverage natural immune signaling pathways, effectively activating APCs. However, their scalability is constrained by the reliance on biological materials, and their limited antigen-loading capacity, combined with rapid clearance *in vivo*, can reduce delivery efficiency 105. Liposomes, versatile carriers capable of encapsulating both hydrophilic and hydrophobic antigens, offer opportunities for functionalization to target specific receptors on APCs. Despite their established biocompatibility and clinical use, liposomes are prone to instability, premature antigen release, and rapid clearance unless further modified, such as through PEGylation 106, 107. Polymer-based systems provide controlled and sustained antigen release, prolonging immune activation. Their flexibility in design allows co-delivery of adjuvants and antigens, but these systems can occasionally elicit unintended immune responses, and their production requires precise fabrication processes 108. Inorganic material-based systems, such as silica or gold nanoparticles, are characterized by high antigen-loading capacity, stability, and multifunctionality, which enables theranostic applications. However, concerns regarding the long-term safety and potential accumulation of non-biodegradable materials pose challenges to their widespread adoption 109. Self-assembling nanoparticles offer a carrier-free approach by forming stable nanostructures from antigens and adjuvants, ensuring high antigen density and reducing the risks associated with synthetic carriers. Nevertheless, their stability in physiological environments and their ability to accommodate diverse antigen types remain key challenges 110. Each of these delivery systems offers distinct strengths and drawbacks, and their application should be guided by the specific antigen properties, the desired immune response, and logistical considerations for manufacturing and deployment. The integration of complementary systems or further optimization may provide inspiring solutions to overcome their respective limitations, advancing the efficacy of cancer immunotherapy.

## Advancing precision oncology with nanovaccines

Nanovaccines represent a promising advancement in cancer immunotherapy, offering a precision-based approach to enhancing therapeutic efficacy while minimizing systemic side effects. Precision therapy involves tailoring treatments to the individual characteristics of a patient's tumor, ensuring that therapies are not only effective but also specific to the tumor's molecular profile. In the context of nanovaccines, precision therapy can be achieved through several mechanisms. Targeted delivery is a cornerstone of nanovaccine precision 53, 111.

By engineering nanocarriers that recognize specific TAAs or tumor endothelial markers, nanovaccines can selectively direct antigens to the tumor site, avoiding off-target effects and enhancing the immune response within the TME. For instance, the HybridDC nanovaccine, incorporating CCR7, tumor antigens, and costimulatory molecules, enhances precise targeting to lymphoid tissues, improving antigen delivery and reshaping the tumor immune landscape. This targeted approach demonstrates robust efficacy and induces long-term memory T cell immunity in glioma models, offering potential for improved cancer immunotherapy 112. Additionally, controlled release mechanisms allow nanovaccines to release their antigenic cargo in response to the unique conditions of the TME, such as low pH, hypoxia, or specific enzymes. Hypoxia-responsive zeolitic imidazolate framework nanoparticles loaded with tirapazamine and resiquimod generate an *in situ* nanovaccine that enhances liver cancer cell killing under hypoxic conditions 113. Furthermore, personalized vaccines mark a pivotal advancement in precision therapy, as they are tailored to the genetic and molecular characteristics of a patient's tumor. Advanced techniques like single cell sequencing enable the identification of patient-specific tumor neoantigens or TAAs for incorporation into nanovaccine design. This personalized approach ensures that the vaccine targets the most relevant and effective antigens, increasing the likelihood of a robust immune response 114, 115. For instance, in a phase I trial, the personalized neoantigen vaccine autogene cevumeran, derived from resected PDAC tumors and based on uridine mRNA-lipoplex nanoparticles, significantly boosted neoantigen-specific T cell responses when administered with atezolizumab and mFOLFIRINOX chemotherapy. This approach notably prolonged recurrence-free survival in PDAC, demonstrating the efficacy of personalized immunotherapy 116. Similarly, in a phase 2b trial, adjuvant therapy combining mRNA-4157, a personalized mRNA-based neoantigen vaccine, with pembrolizumab significantly extended recurrence-free survival in patients with resected high-risk melanoma compared to pembrolizumab alone. This combination, administered to 157 patients, demonstrated a manageable safety profile, supporting the potential of individualized neoantigen therapies in enhancing melanoma treatment outcomes 117. Moreover, personalized nanovaccines can be adjusted for the patient's immune profile, optimizing their immune system's ability to recognize and eliminate cancer cells. A nanochaperone, PBA-nChap, tailors cancer vaccination by capturing and protecting TAAs directly from the tumor site, enhancing their presentation to immune cells. When combined with photodynamic therapy and co-cultured with resected tumor fragments, it synergizes with immune checkpoint blockade to prevent tumor recurrence and metastasis 118.

Nanovaccine technology is transforming precision oncology by addressing the challenge of immunotherapy resistance. This approach not only enhances the effectiveness of existing immunotherapies but also introduces new strategies to overcome resistance, thus advancing the field of cancer treatment towards highly personalized and effective modalities 119. For instance, a therapeutic nanovaccine combining chemotherapy-induced resistant tumor antigens with the TLR 7/8 agonist R848 offers an inspiring approach to overcoming immunotherapy resistance in cancer. This strategy enhances antigen delivery and immune stimulation directly to DCs, boosting their maturation and promoting T cell infiltration while reducing Tregs 120. Similarly, a mesoporous silicon nanovaccine, loaded with antigens from gemcitabine-resistant triple-negative breast cancer cells, shows promise in counteracting drug resistance. This vaccine leverages tumor-specific mutations and antigens to enhance immune activation, improving antigen presentation and T-cell infiltration in tumors. When combined with immunotherapy, this nanovaccine effectively targets resistant tumors, aligning with precision oncology efforts to overcome immunotherapy resistance 121. Additionally, a nanoparticle vaccine platform co-delivering peptide neoantigens and synergistic adjuvants, cGAMP and MPLA, enhances antigen immunogenicity and CD8^+^ T cell activation by targeting DCs and improving lymph node accumulation. This approach delays tumor growth and boosts ICB efficacy in murine models, addressing immunotherapy resistance 122.

Notably, nanovaccines hold immense promise for integration into precision-based multi-modal cancer therapies, offering synergistic enhancements to existing treatments such as chemotherapy, radiotherapy, and targeted therapies. For instance, IVCI nanovaccines, derived from cell lysates treated with ultrahigh doses of chemotherapeutics, enhance chemoimmunotherapy by increasing the abundance and diversity of DAMPs and neoantigens. These nanovaccines, tested in CT26 tumor-bearing mice, substantially boost CD4^+^/CD8^+^ T-cell responses when used alongside immune checkpoint inhibitors 123. Additionally, a peptide-based nanovaccine, generated via enzyme-induced self-assembly alongside ICD from radiotherapy, forms fibrous nanostructures that encapsulate autologous tumor antigens. This strategy enhances antigen presentation in lymph nodes and promotes T cell-mediated immunity by reprogramming the TME, reducing immunosuppressive cells, and boosting the efficacy of radiotherapy 124. Similarly, a specific MnO_2_ nanovaccine, functionalized with mannose, targets innate immune cells to activate the STING pathway, enhancing radiotherapy-induced immune responses. This targeted approach not only inhibits tumor growth and metastasis locally and distantly but also allows for magnetic resonance imaging of nanovaccine distribution *in vivo*, providing a dual-functional strategy for optimized radioimmunotherapy 125. Moreover, a biomimetic nanovaccine co-delivering IL-15 and tumor-associated antigens selectively targets IL-15 to antigen-specific CTLs, reducing off-target toxicity and extending the cytokine's half-life by 8.2-fold. By integrating multivalent IL-15 self-presentation with antigen-specific immune activation, this approach enhances T cell responses and reshapes the TME 126.

## Challenges and future prospects

Nanovaccines represent a promising advancement in enhancing CD8⁺ T cell-based cancer immunotherapy by harnessing the unique properties of nanomaterials to optimize antigen delivery, presentation, and immune activation. Nonetheless, their translation from preclinical research to clinical application is hindered by significant scientific, technical, and regulatory challenges that must be addressed to unlock their full therapeutic potential.

One of the primary obstacles in advancing nanovaccines is the efficient and targeted delivery of antigens to elicit a potent CD8^+^ T cell response. Nanoparticles must navigate biological barriers such as the mononuclear phagocyte system, which often clears them before they reach the intended target. Additionally, nanoparticles face challenges posed by the vascular and endothelial barriers, the dense extracellular matrix, and immune clearance mechanisms. Overcoming these barriers is essential for improving the efficiency and specificity of nanovaccine-based therapies. Developing shape-shifting nanoparticles represents a promising strategy to address these challenges. For instance, FeFKC, an inspiring adaptive material, undergoes multi-step morphological transformations in response to the TME's pH changes, transitioning from single chains to nanoparticles and nanofibers. This programmable shape-shifting enhances tumor penetration, cellular uptake, and lysosomal escape, significantly improving catalytic efficiency in nanocatalytic tumor therapy 127. The use of vasodilators, such as low-dose VEGF or nitric oxide, can temporarily increase tumor blood vessel permeability, facilitating nanoparticle extravasation into the tumor. Hydralazine, an antihypertensive vasodilator, enhances nanoparticle penetration in desmoplastic tumors by reducing tumor stroma and improving tumor hypoxia 128.

Tumor heterogeneity, arising from genetic, epigenetic, and environmental variations within and between tumor subpopulations, significantly complicates the design of universal nanovaccines. Variations in antigen expression across different tumor regions result in subpopulations that evade immune surveillance, leading to therapeutic resistance and tumor progression 129. Single-antigen-targeted nanovaccines, such as those addressing HER2 or EGFR, may fail to target all tumor cells effectively, particularly in highly heterogeneous cancers. To overcome this challenge, modular nanoparticle platforms capable of co-delivering multiple antigens offer a promising solution. For instance, multi-epitope vaccines demonstrated superior therapeutic activity compared to mono-epitope vaccines, as a combination of multiple immunogenic neoantigens generated stronger CD8^+^ T cell-mediated tumor rejection. In addition, AI technologies might provide offer powerful solutions for identifying personalized antigens or nano-based delivery systems tailored to the unique characteristics of a patient's TME. AI-driven analysis of whole-exome and RNA sequencing data from 120 cancer patients identified 212 immunogenic mutations and 178 neo-peptides, highlighting the predictive power of machine learning for neoantigen prioritization 130. Interestingly, the AI-guided ionizable lipid engineering (AGILE) platform uses deep learning and combinatorial chemistry to optimize ionizable lipids for mRNA delivery. AGILE identifies cell-specific lipid preferences, enabling the rapid design of tailored LNPs for efficient mRNA delivery across diverse cell types, expanding the potential of mRNA therapies 131.

Safety remains a critical consideration, with the long-term biocompatibility of nanoparticles being a key challenge, particularly in terms of their accumulation and clearance from the body. Nanoparticles with prolonged circulation times may not be efficiently excreted, potentially resulting in toxicity due to extended exposure. These challenges are especially critical in the treatment of complex diseases like cancer, where narrow therapeutic windows and the risk of off-target effects can lead to significant adverse outcomes 132. Self-eliminating nanoparticles provide a promising solution by enabling targeted vaccine delivery, with triggerable functions that enhance therapeutic efficacy while minimizing collateral damage and side effects 133. Additionally, TME-responsive nanoparticles can be engineered to degrade upon exposure to specific signals present in the TME. The redox and light-responsive nanoparticle's-controlled degradation in the TME enhances ROS generation and overcomes defense mechanisms, improving chemodynamic therapy. Degradation triggered by high glutathione and acidity boosts ROS production, while synergizing with photothermal therapy and drug release for selective and effective tumor suppression 134, 135.

The scalability of nanovaccine production for clinical use remains a critical challenge, as overcoming technical and economic barriers is essential for transitioning from small-scale preclinical studies to large-scale clinical application. Issues such as inconsistent particle size, surface charge, and composition variability hinder reproducibility, while the lack of standardized synthesis and characterization methods impedes quantitative comparison and meta-analysis 136. Recent advancements in continuous manufacturing technologies and automated production systems are addressing these challenges, enabling more consistent nanoparticle synthesis and reducing costs. For instance, a universal, purely biological nanovaccine system is developed, integrating three modules: self-assembling nanoparticles, self-catalyzed synthesis of STING agonists, and delivery vectors targeting the cytosolic surveillance system. This system enables efficient nanoparticle assembly and demonstrates excellent immunostimulatory properties and lymph node targeting 137. To ensure regulatory compliance and facilitate clinical translation, a comprehensive, standardized framework for nanovaccine design, testing, and reporting is urgently needed. This framework should include uniform guidelines for synthesis, characterization, clinical trial protocols, and quality control measures, alongside the establishment of multi-center platforms to enhance reproducibility and accelerate the clinical implementation of nanovaccine technologies.

## Conclusions

Cancer nanovaccines represent a significant advancement in precision medicine, offering the potential to revolutionize cancer treatment by targeting the immunosuppressive TME and enhancing CD8^+^ T cell responses. By integrating nanotechnology with immunotherapy, these vaccines address the physical and immunological barriers that have historically limited the effectiveness of traditional therapies. However, challenges such as optimizing delivery mechanisms, ensuring patient safety, and achieving scalable production remain. Continued progress in biomaterials development and the integration of personalized treatment strategies will be critical to fully realizing the potential of nanovaccines. Moving forward, interdisciplinary collaboration and ongoing technological innovation are essential to overcoming these challenges and achieving durable, effective outcomes in cancer immunotherapy.

## Figures and Tables

**Table 1:** Types of adjuvants for activating CD8^+^ T cells

Adjuvant	Active Component	Delivery System	Associated disease	Mechanism of action	Specific CD8^+^ T cell effects	Clinical application	Ref.
TLR agonist	TLR4/9 agonist ND-MPLA/CpG	High-density lipoprotein nanodiscs	Melanoma, HPV-induced cancer	Enhancing humoral response, inducing tumor regression and reducing plasma cholesterol level	Boosting antigen-specific CD8^+^ T cell responses	Potential nanoplatform for immunotherapy	36
	TLR9 agonist D35	LNP	CRC	Suppressing tumor growth through Th1-related gene induction	Enhancing CD8^+^ T cell activation	Effective immunostimulatory drug formulation for immunotherapy	37
	TLR9 agonist CpG-ODN	Au-Si-IONPs	Colon cancer	Promoting oxidative metabolism and long-term survival of CD8^+^ T cells	Enhancing CD8^+^ T cell memory and recalling responses	Boosting immunotherapeutic outcomes in colon cancer	38
	TLR7/8 agonist 522	PLGA	Melanoma	Enhancing dendritic cell activation, co-stimulation, and MHC I-mediated antigen presentation	Boosting CD8^+^ T cell response	Potential delivery system in melanoma treatment	39
	TLR7/8 agonist 522 (or 528)	PLGA	Melanoma, Bladder cancer, Renal adenocarcinoma	Enhancing dendritic cell activation, co-stimulation, and antigen presentation	Boosting antigen-specific CD8^+^ T cell expansion and potency	Potential as potent immunostimulatory adjuvants in cancer immunotherapy	40
	Poly(I:C) t-TLR7/8a	K-nanoadjuvant	Melanoma, Cervical cancer, Breast cancer	Enhancing dendritic cell efficacy, IL-12 production and natural killer cell responses	Robusting CD8^+^ T cell responses	Augmenting anti-PD1 therapy effectiveness	41
	TLR7/8 agonist R848	Self-immolating nanoadjuvant	Pancreatic cancer	Inducing immunogenic cell death, releasing R848 to enhance dendritic cell activation	Enhancing CTL recruitment	Enhancing immune checkpoint blockade therapy in Pancreatic cancer	42
Cytoplasmic DNA receptor activators	Mn	M@P@HA nanoparticles	Breast cancer	Inducing ROS, disrupting redox homeostasis, activating the STING pathway	Increasing CD8^+^ T cell infiltration	Potent strategy for broad-spectrum tumor immunotherapy	44
	Mn	MnO@mSiO_2_-iRGD	Melanoma	Activating cGAS-STING pathway and enhancing ROS production	Increasing CTL infiltration	Enhancing αPD-1 immunotherapy in melanoma	45
	Cyclic nucleotides	DNCA/CLD nanocomplex	Solid tumors	Activating STING pathway, inducing IFN-I and chemokines	Enhancing CD8^+^ T cell infiltration and activity	Cancer immunotherapy for melanoma, TNBC, and solid tumors	46
	Cyclic nucleotides	cdG@RMSN-PEG-TA	Breast cancer	Stimulating the secretion of IL-6, IL-1β, and IFN-β along with Ser365 protein expression	Enhancing infiltration of leukocytes	Potential nanoplatform for immunotherapy	47
	PC7A	PC7A NP	Melanoma, HPV-induced cancer	Upregulating CXCL9 expression in myeloid cells and IFN-γ by T cells	Enhancing CTL infiltration, boosting IFN-γ production	Potential of targeted nanovaccine delivery methods in cancer immunotherapy	48
	PC7A	PC7A NP	Melanoma, HPV-induced cancer	Activating STING pathway, leading to regression of advanced solid tumors	Boosting CD8^+^ T cell responses	Improving therapeutic outcome of late stage solid cancers	49
Cytoplasmic RNA receptor activators	5'pppdsRNA	LCP	Colorectal liver metastasis	Activating RIG-I, enhancing antigen presentation, and immunogenic TME transformation	Boosting adaptive CD8^+^ T-cell population	Demonstrating superior efficacy in treating colorectal liver metastasis	50
	poly(I:C)	\	Solid tumors	Boosting IL-2, IFN-γ, CAR-T activity, and reducing MDSC suppression	Increasing IL-2 and IFN-γ production, enhanced tumor cell cytotoxicity	Synergistic approach for enhancing CAR-T cell efficacy in tumor immunotherapy	51
	poly(dA:dT)	\	Cancer	Production of type I interferon	Eliciting adaptive T cell immunity	Demonstrating efficacy in vaccination strategies	52

**Table 2:** Antigen-based nanovaccines in CD8^+^ T cell modulation

Delivery System	Antigenic targets	Diseases	Mechanisms	CD8^+^ T cell effects	Therapeutic efficacy	Ref
HM-NPs	Autologous tumor antigen	Melanoma, breast cancer, bladder cancer	Facilitating DCs maturation through TLR4	Activating CTLs	Suppressing tumors recurrence and metastasis	59
smDV-aCTLA-4	CTLA4	Hepatocellular carcinoma	Enhancing antigen presentation, immune checkpoint suppression	Enhanced infiltration, activation, and cytotoxicity of CD8^+^ T cells	Reduced recurrence, enhanced efficacy	60
LeX-modified liposomes	gp100280-288 peptide	Melanoma	DC-SIGN targeting, DC maturation, cross-presentation	Enhanced IFNγ production	Enhanced DC targeting and CD8^+^ T cell activation	61
SPIONs encapsulated in magnetic liposomes	B16-OVA tumor antigens	Melanoma	Magnetic lymph node targeting, enhancing DC uptake or activation	Enhanced CTL tumor infiltration and IFNγ	Tumor growth inhibition in mice	62
PLGA-PEI nanoparticles	Patient-derived melanoma neoantigens	Melanoma	DC maturation and enhancing antigen presentation via PLGA-PEI/poly(I:C) system	Increased activation of CD8^+^ T cells	Activating specific immune response	63
PEI-PEG nanoparticles coated	Personalized peptides	Melanoma	Enhancing DC uptake, activation and antigen cross-presentation	Robust priming of neoantigen-specific CD8^+^ T cells	Strong suppression of established primary tumors	64
Tumor antigen assembly nanoparticles	NY-ESO-1	Cancer	Endolysosomal escape Enhancing cross-presentation	Enhanced T cell proliferation	Enhanced CD8^+^ T cell responses	65
PLGA nanoparticles	Ovarian cancer cell antigens	Ovarian cancer	FCM encapsulation of dendritic and cancer cells	Enhanced T-cell activation	Delayed tumor growth	66
Poly(I:C) HMW ODN 1826	OVA pTVG-AR	Prostate cancer	Decreasing PD-1 expression on CD8^+^ T cells	Increased tumor-infiltrating antigen-specific CD8^+^ T cells	Greater tumor growth suppression	67
CaP nanoparticles	Hemagglutinin	Colon cancer	Type I interferon-dependent activation	Increased frequency of tumor-infiltrating CD8^+^ T cells	Significant tumor growth control	69
M-M NPs	Whole cell antigens	Melanoma	TME-responsive GSH depletion and ROS generation	Increased tumor-specific CD8^+^ T cells	Suppressed primary tumor growth	70
CPMV-NY-ESO-1	NY-ESO-1_157-165_	TNBC, melanoma, myeloma, OC	Enhanced uptake by APCs	Antigen-specific proliferation	Potential application against NY-ESO-1^+^ malignancies	71
LCCT NPs	TIM-3	Colorectal cancer	Inducing TIM-3 blockade and cross-presentation	Activating effector and memory CD8^+^ T cells	Suppressing cancer relapse and metastasis	72
R-DOTAP	KF18 RF9 MUC1	HPV-related cancers	Stimulating type I IFN production	Generating robust CD8^+^ T cell response	Complete regression of large established tumors	73
DMPC:DMPG:Chol:DOPE liposomes	HER2 P5 peptide PADRE peptide	HER2-positive breast cancer	Increasing IFN-γ production	Enhanced CD4^+^ and CD8^+^ T cell activation	Increased survival	74
Polymeric nanovaccine platform	OVA	Melanoma Colon carcinoma	Enhancing endocytosis by APCs TLR7 activation in endosomes	Enhanced antigen-specific CD8^+^ T cell responses	Increased median survival	75
8FNs	FK-33	B16-F10 tumor	Inducing DCs activation in the lymph nodes	Eliciting CD8^+^ T cell response	Inhibiting tumor metastasis growth	76
PEI-based nanovaccine platform	OVA TSA	Colon carcinoma Melanoma	Enhancing antigen/CpG uptake by dendritic cells	Robust expansion of antigen-specific CD8^+^ T cells	Long-term protection against tumor rechallenges	77
BCMA peptide loaded-NPs	BCMA	Multiple myeloma	Increasing peptide delivery to human DCs	Enhancing induction of BCMA-specific CTLs	Enhancing immunotherapy efficacy	78
PpASE	MHC-I MHC-II	Melanoma	Enhancing antigen uptake by APCs	Simultaneous activation of CD8^+^ and CD4^+^ T cells	Significant tumor growth inhibition	79
Mn4^+^-SNPs	HPV16 E7-derived peptide GF001	HPV-related tumors	Enhancing inflammatory pathways Efficient antigen delivery	Robust CD8^+^ T cell responses Enhanced cytotoxic activity	Achieved remission in HPV tumor	80
Mesoporous silica nanoparticles	OVA	Cancer	DC-specific targeting via TY peptide	Enhanced antigen-specific CD8^+^ T cell activation Increased CTL priming	Enhanced tumor elimination	81
SNP-7/8a	Diverse peptide neoantigens	Cancer	Enhancing APC uptake and activation	Enhanced CD8^+^ T cell immunity	Improved tumor clearance in mice and primates	82
Self-assembling peptide-TLR2 agonist conjugates	MAGE-A1 peptide	Cancer	TLR2-mediated immune activation	Enhanced CD8^+^ T cell activation	Enhanced antitumor response	83
Self-assembled TLR7/8 agonist-epitope conjugates	Survivin (Sur) MAGE-1 gp100	Melanoma	MyD88-dependent TLR signaling activation	Enhanced CD8^+^ T cell activation Increased memory T cell responses	Enhanced anti-tumor immunity	84
CNPs	SIINFEKL peptide	Pancreatic ductal adenocarcinoma	Enhancing antigen presentation via MHC	Activation of SIINFEKL-specific CD8^+^ OT-1 T cells	Effective lysis of Panc-OVA cells	85
PEGylated PLGA nanoparticles	OVA	\	Enhancing DC targeting via surface receptors	Enhanced proliferation Increased IFN-γ production	All targeted vaccines superior to non-targeted	86
PLGA	OVA	Cancer	PLGA/OVA NPs taken up by Gr-1high cells	Activating antigen-specific CD8^+^ T cells	Enhanced antitumor response	87
Self-adjuvanting polymeric nanocapsules	OVA	Lymphoma	Endosomal escape via C7A protonation	Increased proliferation	Enhanced immune memory formation	88
PGA-IMDQ nanoparticles	OVA	Lymphoma	Redox-triggered antigen/adjuvant release	Increased tumor-infiltrating CD8^+^ T cells	Significant tumor growth inhibition	89
Magnetic nanoparticles	OVA254-267 peptide	Melanoma	Enhancing DC activation Improving antigen presentation	Enhanced CD8^+^ T cell activation	Anti-melanoma activity	90
AuNPs	SIINFEKL peptide	Infectious diseases Cancer	Efficient DC uptake and internalization	High IFN-γ and TNF production Strong antigen-specific CD8^+^ T cell proliferation	Platform potentially applicable to infectious diseases and cancer	91
IO-LPMONs	OVA	Cancer	Activating dendritic cells	Activating CD4^+^ and CD8^+^ T cells	Demonstrating significant combined antitumor effects	92
Encapsulin	OT-1 peptide of OVA	Melanoma	Enhancing presentation effect of OT-1 peptides via DCs	Activating OT-1 peptide specific cytotoxic CD8^+^T cells	Suppressing tumor growth	93
VLPs	OVA_B_peptide and OVA_T_peptide	Cancer	Inducing highly effective cross-presentation	Increasing the proportions of CD8^+^T cells and activating a T epitope-specific CTL response	Inhibiting tumor growth in mouse tumor model	94
I-OVA NE	I-OVA, IKVAV and OVA_257-264_epitope conjugated peptide.	Cancer	Promoting antigen uptake of I-OVA NE and prolonging the nasal residence time	Enhancing CTL activity and Th1 immune response	Induction of protective immunity in E.G7-OVA tumor-bearing mice	95

**Table 3:** Nucleic acid-based nanovaccines in CD8^+^ T cell modulation

Delivery System	Antigenic targets	Associated disease	Mechanism of action	Specific CD8^+^ T cell effects	Clinical application	Ref.
E749-57-HSP110-RGD	HPV16 E7(49-57) epitope	Cervical cancer	Stimulating robust CD8^+^ T cell activation and IFN-γ production	Boosting CD8^+^ T cell activation	Potential therapeutic role in cervical cancer immunotherapy	96
Alg-Tat-gp100	gp100	Melanoma	Boosting IFN-γ secretion and cytotoxic T cell activation	Enhancing cytotoxic T cell activation	Enhancing melanoma immunotherapy efficacy	97
DLnano-vaccines	Gp100 and Trp2	Melanoma	Enhancing CD8^+^ T cell responses and tumor control	Eliciting strong CTL responses	Adjunct therapy with oxaliplatin	98
NPs	MUC1	TNBC	Suppressing myeloid-derived suppressor cells, inhibitnig STAT3 and pro-tumorigenic cytokines, and promoting tumor cell apoptosis	Decreasing regulatory T cells, enhancing CTL responses	Potential therapeutic role in TNBC immunotherapy	99
LNPs	Gp100 and TRP2	Melanoma	Tumor shrinkage and activation of CD8^+^ T cells	Inducing cytotoxic CD8^+^ T cell responses	Potential therapeutic role in cancer immunotherapy	101
LNPs	HPV-E7	HPV-positive OSCC	Activating CD8^+^ T cells and modulating their functional commitment in TME, promoting tumor regression	Enhancing HPV-specific CD8^+^ T cell responses	Potential combination therapy with ICIs	103
